# Adalimumab Therapy Restores the Gut Microbiota in Patients With Ankylosing Spondylitis

**DOI:** 10.3389/fimmu.2021.700570

**Published:** 2021-09-01

**Authors:** Zena Chen, Xuqi Zheng, Xinyu Wu, Jialing Wu, Xiaomin Li, Qiujing Wei, Xi Zhang, Linkai Fang, Ou Jin, Jieruo Gu

**Affiliations:** Rheumatology and Immunology Department, Third Affiliated Hospital of Sun Yat-sen University, Guangzhou, China

**Keywords:** adalimumab, TNF, gut microbiome, ankylosing spondylitis, biomarker

## Abstract

Growing evidence suggests that the gut microbiota is involved in the initiation and progression of ankylosing spondylitis (AS). In this study, we aimed to explore the gut microbiome alterations during adalimumab therapy and verify microbiome biomarkers predicting treatment response. By evaluating the gut microbial features of 30 AS patients before and after adalimumab therapy for 6 months and 24 healthy controls, we confirmed that the microbiome was restored remarkably after 6 months of adalimumab therapy in AS patients. We then compared the baseline gut microbiome of 22 adalimumab responders with 8 non-responders, a higher abundance of *Comamonas* was revealed in the latter, although no statistical difference was found after adjusting for the false discovery rate. These results suggested that adalimumab therapy restored the gut microbiome in AS patients and indicated the utility of gut microbiome to be potential biomarkers for therapeutic evaluation. These findings provided an insight into the development of predictive tools and the establishment of precise medical interventions for clinical practice.

## Introduction

Ankylosing spondylitis (AS) is a chronic inflammatory disorder that affects the axial skeleton, causing characteristic inflammatory back pain that can lead to structural and functional impairments and a decreased quality of life (1). Among patients with AS, 40–60% present with subclinical intestinal inflammation, and 5–10% progress to clinical inflammatory bowel disease (IBD) (2). In recent years, substantial evidence has indicated the vital role of the gut microbiota in the initiation and progression of IBD (3). Similarly, a growing number of studies revealed perturbed gut microbiota in AS or spondyloarthritis (SpA) patients (4), and also in AS animal model typically HLA-B27 transgenic rats (5). Moreover, some altered species like *Dialister*, was related to AS disease activity (6). Recently, HLA- B27- positive healthy individuals were identified with a significantly different microbiome (7), indicating that the gut microbiota may in fact be a driver of AS.

The development of tumor necrosis factor inhibitor (TNFi) and its introduction into clinics was a milestone in the treatments for autoimmune diseases, including AS, IBD, psoriasis, and rheumatoid arthritis. It is well known that TNFi improves symptoms and inflammatory cytokine levels in patients with AS. However, it is unknown whether TNFi affects the gut microbiome due to a lack of evidence. A previous study analyzed the gut microbiome of SpA patients before and after 3 months of TNFi treatment, using stool samples (8). Only a modest difference in the alpha diversity of the gut microbiome was found, while no specific bacterial taxa were observed. But this study did not include healthy controls, so it is uncertain whether TNFi treatment was responsible for restoring the gut microbiome to a healthier status. Recently, restoration of gut microbiota composition was revealed in proteoglycan-induced AS mice after TNFi treatment (9), indicating that TNFi treatment might affect the gut microbiota.

As humanized monoclonal antibody targeting TNF, adalimumab has been successfully used to manage inflammation in AS patients in clinic, especially in those with extra-articular symptoms such as IBD and uveitis (10). It was also reported to improve symptoms and restore gut microbiota in patients with IBD (11). In addition, baseline features of the gut microbiome were valuable in predicting the treatment response to TNFi in patients with IBD (12). However, the association between the gut microbiome alteration and adalimumab treatment in AS patients is unknown.

In this study, we recruited AS patients prescribed adalimumab and healthy controls to evaluate the effect of adalimumab treatment on the gut microbiome. Further, we explored whether the gut microbiome can be used to predict the response to adalimumab treatment in AS patients.

## Materials and Methods

### Populations and Sample Size

We conducted a prospective observational study between January 2017 and July 2018. Patients with AS who were initiated adalimumab therapy were consecutively recruited at the outpatient clinic of Rheumatology at the Third Affiliated Hospital of Sun Yat-sen University. The inclusion criteria were as follows: (1) age of 18 years or older, (2) fulfillment of the 1984 modified New York Criteria for AS (13), (3) inadequate improvement despite taking at least two non-steroidal anti-inflammatory drugs (NSAIDs) for 4 weeks, (4) Bath Ankylosing Spondylitis Disease Activity Index (BASDAI) > 4 at baseline, and (5) no TNFi treatment in the 6 months prior to recruitment. The exclusion criteria were as follows: (1) antibiotic or probiotic treatment within 2 months of fecal collection, and (2) chronic infectious disease such as tuberculosis or hepatitis B. Fecal samples and clinical data, including demographic information, disease-related characteristics, and measurements of disease activity or functional status, were collected by trained investigators at baseline (M0) and after 6 months of adalimumab treatment (M6). Disease activity of AS was assessed using the Ankylosing Spondylitis Disease Activity Score (ASDAS) according to its cutoff points, while therapeutic response was defined using the change in ASDAS (△ASDAS) as reported previously (14, 15). Clinical response was defined as ASDAS change > 1.1, and no clinical response was defined as ASDAS change ≤ 1.1. In order to determine the suitable sample size, we hypothesized that, after treatment with adalimumab, 50-60% of patients would experience an alteration in the abundance of potentially disease-related components of gut microbiota (16). A total sample size of at least 13 pairs of AS patients (pre- and post-treatment) was required to achieve a power of 90% and a two-sided significance of 5%. The sample size was estimated by PASS 15 software (https://www.ncss.com).

Healthy controls were recruited from volunteers, at the same hospital, who had not been diagnosed with AS, other rheumatic diseases, or chronic infectious diseases, and who had not received antibiotic or probiotic treatment within 2 months of fecal collection.

This study was conducted in compliance with the Declaration of Helsinki and was approved by the ethics committee of the Third Affiliated Hospital of Sun Yat-sen University. Before enrollment, written informed consent was obtained from all subjects for research and publication of their data.

### Fecal Sample Collection

Fresh fecal samples were collected from the patients at baseline and at 6 months, and from healthy controls at baseline. After defecation fecal samples were collected and immediately placed on dry ice, and then transferred to the laboratory within 2 hours, followed by the storage at -80°C until DNA extraction.

### DNA Extraction and 16S rRNA Gene Sequencing

Microbial genomic DNA was extracted from fecal samples using a DNA isolation kit (Tiangen Biotech Co., Ltd., Beijing, China) according to the manufacturer’s instructions. The V4 region of the 16S rRNA gene from each sample was amplified by polymerase chain reaction (PCR) using specific primers (515F, 5’-GTGCCAGCMGCCGCGGTAA-3’, and 806R, 5’-GGACTACHVGGGTWTCTAAT-3’) with barcodes (17). After purification, the DNA library was obtained and sequenced using the Illumina Hiseq2500 platform to generate 250 bp paired-end reads.

The reads were purified and merged and then processed using a QIIME-based bioinformatics pipeline (v1.9.1) (18). Briefly, we curated the sequences to reduce sequencing and PCR errors, aligned the resulting sequences to the SILVA 16S rRNA sequence database, and used UCHIME to remove any chimeric sequences as per to the GOLD database. Sequences were clustered into operational taxonomic units (OTUs) with a 97% similarity cutoff using the average neighbor algorithm. All sequences were classified using a naïve Bayesian classifier trained on the RDP training set, and the OTUs were assigned a classification based on the taxonomy with the majority consensus of sequences within a given OTU at a threshold of 80%. We obtained the OTU table and taxonomy tree, and further analysis of the α- and β-diversity indices was conducted using Microbiomeanalyst (19). It is a web-based tool for comprehensive statistical, visual and meta-analysis of microbiome data.

### Statistical Analysis

Graphpad 8.0 (IBM, USA) and R (version 3.4.3) were used to analyze the data. Longitudinal comparisons were used to analyze changes in patients after treatment, and cross-sectional comparisons were made between patients and healthy controls.

Gut microbiome composition was represented by α- and β-diversity. To assess α-diversity (20), the Shannon and Simpson indices were calculated for each sample in the dataset. Wilcoxon rank sum tests were performed for pairwise comparisons within patients with AS, and Mann-Whitney tests were performed for comparisons between patients and healthy controls. To measure β-diversity, the UniFrac distance between samples was calculated (21), and permutational multivariate analysis of variance using distance matrices (PERMANOVA) was used to assess the overlap of taxonomy between patients with AS and healthy controls. Comparisons of the relative abundance of taxa were made using Wilcoxon rank sum or Mann-Whitney tests. A *P* value of less than 0.05, after correcting for false discovery rate (FDR) for multiple comparisons, was considered statistically significant.

## Results

### Clinical Characteristic of AS Patients

A total of 30 patients (mean age, 31.23 ± 7.48 years) and 24 healthy controls (mean age, 38.54 ± 10.79 years) were obtained in this study. All included patients were HLA-B27 positive, 12 of them (40%) had peripheral arthritis and 5 (17%) had a positive family history. None of these 30 AS patients had established IBD, psoriasis, or uveitis history. Before treatment, 15 patients had a high level of disease activity and 13 patients had very high level of disease activity. NSAIDs and sulfasalazine (SASP) were prescribed for 20 and 5 patients respectively. During adalimumab therapy, NSAIDs and SASP were remained for those patients.

Overall, clinical symptoms and signs of AS patients were greatly relieved after 6 months of adalimumab therapy, along with improvements in C-reactive protein (CRP), erythrocyte sedimentation rate (ESR), BASDAI, ASDAS, Bath Ankylosing Spondylitis Functional Index (BASFI), and Bath Ankylosing Spondylitis Metrology Index (BASMI), comparing with baseline values (all *P* values < 0.01, **Table 1**). Six patients still had high or very high levels of disease activity even after treatment. As for treatment response, 22 patients responded to adalimumab therapy (△ASDAS > 1.1, R) while 8 patients did not (△ASDAS ≤ 1.1, NR). Patients exhibiting different clinical responses were similar in terms of sex, age, and disease activity before treatment (**Table S1**).

**Table 1:** Clinical characteristic of AS patients.

	M0	M6
Age (years)	31.23 ± 7.48	
Males (n, %)	27 (90)	
Duration (years)^§^	10 (5)	
NSAIDs (n, %)	20 (66.7)	
SASP (n, %)	5 (16.7)	
CRP (mg/L)^§^	11.25 (21.70)	0.95 (4.25)***
ESR (mm/h)^§^	14.00 (25.50)	4.50 (6.25)***
BASDAI^§^	5.23 (1.51)	2.23 (2.72)***
BASFI^§^	3.72 (2.64)	1.60 (2.35)***
BASMI^§^	2.50 (3.00)	1.00 (3.00)**
ASDAS^§^	3.43 (1.29)	1.33 (1.16)***
Inactive^†^	0	14
Low activity^†^	2	10
High activity^†^	15	5
Very high activity^†^	13	1
BASDAI > 4^†^	30	4

^§^Data expressed as median (IQR); ^†^data expressed as frequency. Comparisons of CRP, ESR, BASDAI, BASFI, BASMI, and ASDAS between M0 and M6 were calculated using Wilcoxon rank-sum tests. **P ＜ 0.01, ***P ＜ 0.001. AS, ankylosing spondylitis; IQR, interquartile range; NSAIDs, non-steroidal anti-inflammatory drugs; SASP, sulfasalazine; CRP, C-reactive protein; ESR, erythrocyte sedimentation rate; BASDAI, Bath Ankylosing Spondylitis Disease Activity Index; BASFI, Bath Ankylosing Spondylitis Functional Index; BASMI, Bath Ankylosing Spondylitis Metrology Index; ASDAS, ankylosing spondylitis disease activity score; M0, baseline; M6, after 6 months of treatment.

### Association Between Gut Microbiome and Adalimumab Therapy

In the present study, an average of 65 194 (range, 45 295–78 205) high-quality effective reads were obtained from 84 samples. After taxonomic identification and filtering out taxa with a very low abundance (reads had to be present in at least 20% of the samples, with a count of more than 2), 602 OTUs remained for further analysis. After species annotation, a total of 9 phyla, 16 classes, 24 orders, 47 families, and 117 genera were obtained.

**Figure 1:**
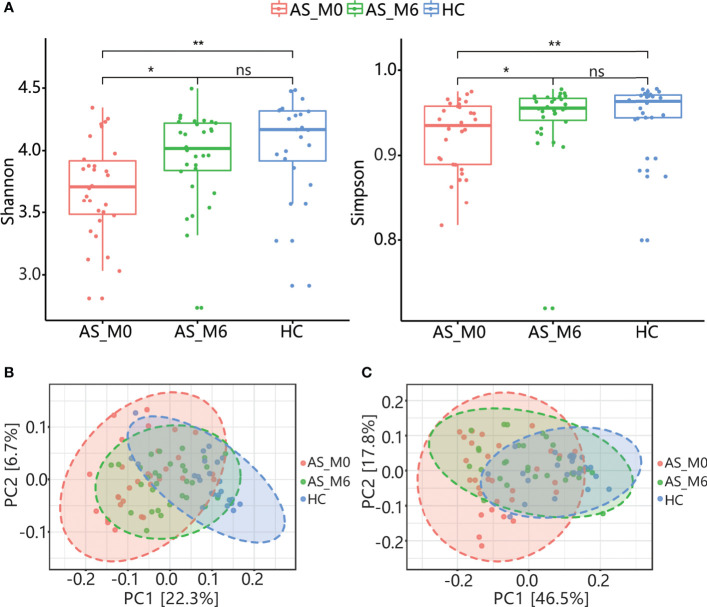
Community structure of gut microbiota in AS patients and HCs. **(A)** α-diversity of gut microbiota (Shannon and Simpson index) among AS patients at baseline and after treatment and among HCs. The horizontal bar within each box represents the median. The bottom and top of each box represent the 25^th^ and 75^th^ percentiles, respectively. The upper and lower whiskers extend to data no more than 1.5 × the IQR from the upper and lower edges of the box, respectively. *P < 0.05, **P < 0.01, ns, *P* > 0.05. PCoA plot based on the unweighted UniFrac distance **(B)** and weighted UniFrac distance **(C)** of gut microbiota from AS patients at baseline and after treatment and from HCs. AS, ankylosing spondylitis; AS_M0, AS patients at baseline; AS_M6, AS patients after treatment; HC, healthy control; IQR, interquartile range; PCoA, principal coordinates analysis.

For α-diversity, the Shannon and Simpson indices of AS patients before treatment (AS_M0) were obviously lower than healthy controls (*P*<0.01, **Figure 1A**), and there were no statistical differences of the above indices between two groups after treatment (*P*>0.05, **Figure 1A**). Similarly, noteworthy difference in β-diversity between the two groups was found before treatment (*P* < 0.001), while no statistical difference was found after treatment, according to the results of PERMANOVA based on weighted and unweighted UniFrac distance (**Figures 1B, C**). These results revealed an alteration of gut microbial community structure in AS patients and an impact of adalimumab treatment on the gut microbiota.

The three most dominant phyla were *Firmicutes*, *Bacteroidetes*, and *Actinobacteria*, which accounted for over 95% of total abundance both in AS patients and healthy controls. The top fifteen relative abundances of taxa at different taxonomic levels in patients with AS and in healthy controls are listed in **Table S2**.

**Figure 2:**
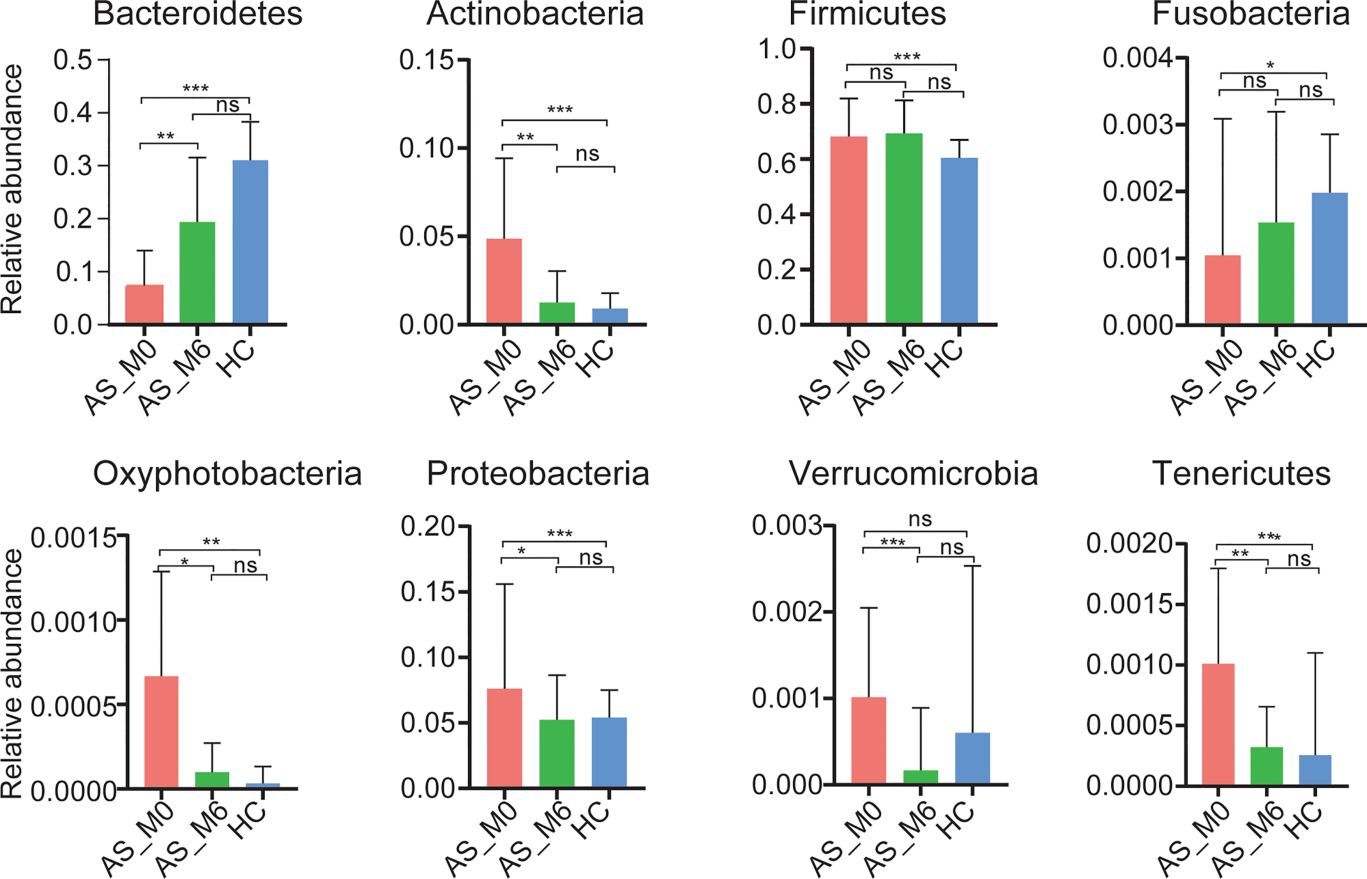
Relative abundance of phyla in different groups. Bars represent the mean ± SEM. *P < 0.05, **P < 0.01, ***P < 0.001, ns, *P* > 0.05.

**Figure 3:**
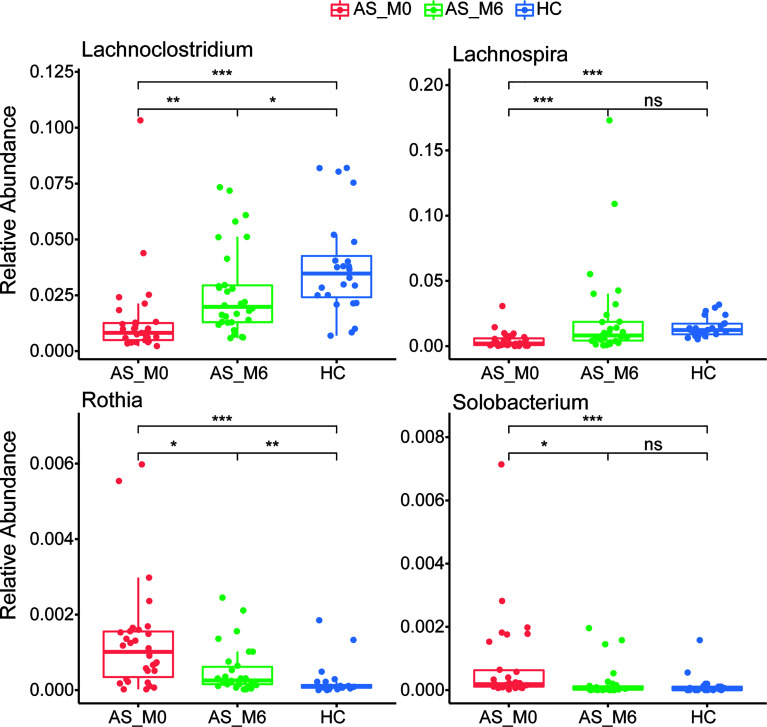
Differential relative abundance of genera in AS patients before and after treatment. AS, ankylosing spondylitis. The horizontal bar within each box represents the median. The bottom and top of each box represent the 25^th^ and 75^th^ percentiles, respectively. The upper and lower whiskers extend to data no more than 1.5 × the IQR from the upper and lower edges of the box, respectively. *P < 0.05, **P < 0.01, ***P < 0.001, ns, *P* > 0.05.

At the phylum level, gut microbiota from AS patients before treatment exhibited a significantly higher abundance of *Actinobacteria*, *Firmicutes*, *Oxyphotobacteria*, *Preteobacteria*, and *Tenericutes* (all *P <*0.01, **Figure 2**), as well as a lower abundance of *Bacteroidetes* and *Fusobacteria* than the microbiota from healthy controls (*P <*0.001 and *P <*0.05, respectively, **Figure 2**). Interestingly, the relative abundance of these seven phyla shifted during adalimumab therapy, eventually resulting in no statistical difference between the two groups (all *P >*0.05, **Figure 2**). At the genus level, 73 genera with distinguishing abundance (such as *Bacteroides*, *Megamonas*, and *Collinsella*) were identified in AS patients before treatment compared with healthy controls (all *P*<0.05, **Table S3**). Relative abundance of these genera altered during adalimumab therapy, and in particular four genera namely *Lachnoclostridium*, *Lachnospira*, *Solobacterium*, and *Rothia* shifted greatly during this time (all *P*<0.05, **Figure 3** and **Table S3**). The relative abundance of *Lachnoclostridium*, *Lachnospira*, and *Solobacterium* in AS patients was restored to levels similar with healthy controls after therapy (*P*>0.05, **Figure 3** and **Table S3**). Only 28 out of the 73 genera (such as *Rothia, Streptococcus, Blautia*, and *Dorea*) remained statistically different (all *P*<0.05, **Table S3**) between the two groups after therapy.

### Association Between Gut Microbiome and Response to Adalimumab Therapy

**Figure 4:**
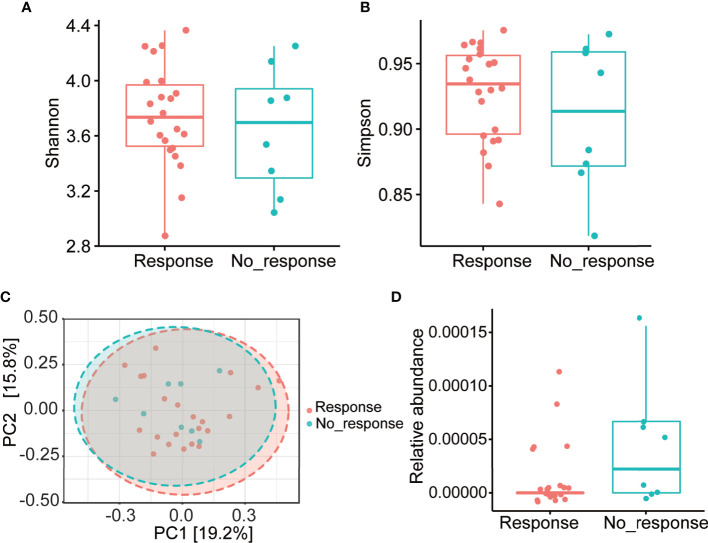
Gut microbial community structure of AS patients before treatment with different responses to Adalimumab. **(A, B)** α-diversity; **(C)** PCoA based on unweighted UniFrac distance. **(D)** Relative abundance of *Comamonas* in response and no_response subgroups. AS, ankylosing spondylitis; PCoA, principal coordinates analysis.

We further investigated whether the gut microbial community structure in patients with AS before adalimumab therapy was related to the treatment response. No significant differences of clinical status as well as α- and β-diversity were found between the responders and non-responders (**Table S1** and **Figures 4A–C**). The abundance of microbiota at different taxonomic levels were then analyzed and no differences were found at the phylum, class, order, or family levels, either. At the genus level, a higher abundance of *Comamonas* was observed in the non-responder group (**Figure 4D**). Unfortunately, after adjusting for FDR, no statistical difference was found.

## Discussion

Ankylosing spondylitis is a chronic inflammatory disease that is thought to be associated with the gut microbiota. Previous studies have suggested that TNFi therapy improves the gut microbial community. In the current study, we illustrated the effect of adalimumab on the gut microbiota composition in AS patients during 6 months of treatment. We observed that the overall gut microbial composition of AS patients exhibited obvious differences comparing with healthy controls, as did most bacterial taxa. After 6 months of adalimumab treatment, the gut microbial composition was restored to a state similar with healthy controls. In addition, no difference was observed in the overall gut composition between the responders and non-responders in AS patients. A higher abundance of *Comamonas* was observed in the non-responders, but this result was not statistically significant after adjustment.

Decreased gut microbiota diversity occurs in many diseases, including IBD and AS. In the current study, we confirmed that gut microbiota diversity was reduced in AS. In addition, we identified bacterial species which were differentiated between AS patients and controls. Depletion of *Bacteroides* and *Megamonas* and enrichment of *Collinsella* in AS patients were noted in our study, which was consistent with findings of previous studies (22, 23). All of these three species restored to indistinguishable from healthy controls after treatment. Carriage of *Dialister* species has been previously reported be associated with disease activity in SpA patients (6). However, we found depletion of *Dialister* in AS patients before treatment and it was restored to similar with healthy controls after treatment. Neither Yin et al. (16) revealed remarkable difference of *Dialister* in AS patients. So, the pathogenic significance of *Dialister* is therefore uncertain.

Restoration of the gut microbiota after treatment has been reported in several other autoimmune diseases, such as IBD and RA (24). One previous study analyzed patients with SpA before and after TNFi therapy and revealed modest differences in microbial composition, but no specific taxon was found to be modulated, which is likely due to the small sample size (8). In another previous study comparing AS patient with and without TNFi treatment, restoration of the overall microbial composition and the abundance of specific taxa were both revealed (16). These indicated TNFi therapy was correlated with a restoration of the perturbed gut microbiota. However, the previous studies did not assess the change in the gut microbiota dynamics of each individual patient. In our study, we compared the baseline state of the gut microbiome of each patient to that 6 months after treatment. Adalimumab therapy was associated with restoration of the microbial composition, and several notable bacterial species modulated by the treatment were identified. We observed that adalimumab therapy restored the normal abundances of *Bacteroidetes* and *Firmicutes*. A decrease in the *Bacteroidetes/Firmicutes* ratio has been associated with autoimmune diseases. Moreover, at the genus level, the abundance of four genera were found to shifted greatly during treatment. Specifically, *Lachnoclostridium* was reduced in AS patients and increased to a level similar with healthy controls after treatment. *Lachnoclostridium* was also found to be less abundant in children with autism spectrum disorders in a previous study (25). As known, AS patients are prone to mental disorders such as depression (26), the effect of perturbations in the gut microbiome on mental disorders in AS patients is worth investigation.

Although great improvement has been reported in some AS patients treated with TNFi, nearly 30% of patients show no response. In the current study, we observed a comparable proportion that 8 out of 30 enrolled patients showed no response to TNFi therapy Thus it is important to identify non-responsive patients, as TNFi therapy is costly. An increasing number of studies have indicated that the gut microbiome was a potential indicator of the response to TNFi treatment, and gut microbiome plays an important role in drug efficacy (27–29). To our knowledge, only one previous study has utilized gut microbiome features to predict the TNFi response in SpA patients in which only 8 non-responders and 5 responders were included (8). A higher abundance of *Burkholderiales* orders was observed in responders prior to treatment. In our study, we included a larger cohort and longer treatment durations and a higher abundance of the *Comamonas* genus in non-responders to adalimumab prior to treatment were revealed. *Comamonas* species are infrequently reported as an infectious agent in routine clinical practice due to rare isolates in microbiology laboratories. In recent years, *Comamonas kerstersii and Comamonas testosteroni* were identified to cause appendicitis and bacteremia by microbiome sequencing (30, 31). Subclinical gut inflammation is common in AS patients, but its relationship with *Comamonas* needs further study. The discrepancy between our results and those of the prior study may be due to differences in racial composition, dietary habits, and treatment duration. These two studies together suggest a possible association between gut microbial features and clinical response, without presuming causality. If the results are further confirmed, it could be clinically helpful to use gut microbiome features as a reliable indicator of treatment response before the initiation of TNFi therapy to avoid a delay in symptom relief and ease the economic burden on health services, paving the way for precision therapy for AS.

Our study had several limitations. Firstly, patients treated with other TNFis or other treatments such as NSAIDs were not recruited, so we cannot conclude that the restoration of the microbiota was specifically related to adalimumab treatment. Secondly, as information of dietary patterns was not collected, the influence of diet was not taken into account. However, since we compared the same patients before and after treatment, any effects of diet should have been somewhat reduced. Finally, due to the small size of our cohort, the power of the statistical analysis may be limited. Nevertheless, in our study, nearly 30% of the patients showed no response to adalimumab therapy, which was consistent with previous larger-scale studies, thereby enhancing the external validity of our study. Further prospective, large-scale studies are required to confirm our results.

## Conclusion

In summary, our study revealed the gut microbiota features in patients with AS and provided insights into the dynamic alterations during adalimumab treatment. Our investigation suggests that the gut microbiota may be a potential tool for predicting the treatment response to adalimumab in AS patients. Additional studies are needed to further investigate the exact bacterial species that play key roles in the response to adalimumab treatment, which will be helpful to achieve precise medical interventions.

## Data Availability Statement

The data presented in the study are deposited in the SRA repository, accession number is PRJNA755445.

## Ethics Statement

The studies involving human participants were reviewed and approved by Ethics committee of the Third Affiliated Hospital of Sun Yat-sen University. The patients/participants provided their written informed consent to participate in this study.

## Author Contributions

(I) Conception and design: JG and ZC. (II) Administrative support: JG. (III) Provision of study materials or patients: All authors. (IV) Collection and assembly of data: ZC. (V) Data analysis and interpretation: All authors. (VI) Manuscript writing and revision: All authors. (VII) Final approval of the manuscript: All authors. (VIII) Funding acquisition: JG. All authors contributed to the article and approved the submitted version.

## Funding

This study was supported by the General Program of the National Natural Science Foundation of China (81871294) and the Science and Technology Planning Project of Guangdong Province (2019B030316004).

## Conflict of Interest

The authors declare that the research was conducted in the absence of any commercial or financial relationships that could be construed as a potential conflict of interest.

The reviewer WS declared a shared affiliation with the authors to the handling editor at the time of review.

## Publisher’s Note

All claims expressed in this article are solely those of the authors and do not necessarily represent those of their affiliated organizations, or those of the publisher, the editors and the reviewers. Any product that may be evaluated in this article, or claim that may be made by its manufacturer, is not guaranteed or endorsed by the publisher.
