# Supplementary material for: Adalimumab Therapy Restores the Gut Microbiota in Patients With Ankylosing Spondylitis
Source: Front Immunol. 2021 Sep 1;12:700570. doi: 10.3389/fimmu.2021.700570 (PMC8441001; doi:10.3389/fimmu.2021.700570)
Supplement: Supplementary file 1 [file Table_1.docx]

**Supplementary**

**Table S1**. Baseline characteristic of AS patients with different response to treatment

|  | **No response (n=8)** | **Response (n=22)** | ***P* value** |
| --- | --- | --- | --- |
| Male | 7 | 20 | .600 |
| Age (years) | 30.5 (16.25) | 30 (7) | .933 |
| BMI (kg/m2) | 20.73 (8.91) | 21.72 (5.79) | .880 |
| CRP (mg/L) | 9.4 (26.65) | 8 (12.2) | .132 |
| ESR (mm/h) | 11 (36) | 12 (12) | .326 |
| BASDAI | 5.19 (2.15) | 4.61 (1.64) | .393 |
| BASFI | 3.05 (3.46) | 3.51 (2.6) | .959 |
| BASMI | 4 (4.5) | 3 (4) | .221 |
| ASDAS | 3.32 (1.74) | 3.12 (1.52) | .074 |
| Disease status | | | |
| Inactive | 0 | 0 | 0.227 |
| Low activity | 0 | 2 |  |
| High activity | 5 | 10 |  |
| Very high activity | 3 | 10 |  |

Data were expressed as median (IQR) or frequency.

**Table S2.** The relative abundance of top 15 taxa at different taxonomic levels

| Taxonomy | AS_M0 | AS_M6 | HC |
| --- | --- | --- | --- |
| Phylum |  |  |  |
| Firmicutes | 0.622 | 0.676 | 0.709 |
| Bacteroidetes | 0.141 | 0.120 | 0.171 |
| Actinobacteria | 0.111 | 0.095 | 0.046 |
| Proteobacteria | 0.089 | 0.067 | 0.045 |
| Verrucomicrobia | 0.026 | 0.027 | 0.009 |
| Fusobacteria | 0.008 | 0.011 | 0.015 |
| Tenericutes | 0.001 | 0.004 | 0.001 |
| unidentified_Bacteria | 0.000 | 0.001 | 0.003 |
| Oxyphotobacteria | 0.001 | 0.001 | 0.000 |
| Class |  |  |  |
| Clostridia | 0.444 | 0.513 | 0.544 |
| Bacteroidia | 0.141 | 0.120 | 0.171 |
| Negativicutes | 0.122 | 0.107 | 0.118 |
| unidentified_Actinobacteria | 0.105 | 0.089 | 0.036 |
| Gammaproteobacteria | 0.087 | 0.064 | 0.037 |
| Erysipelotrichia | 0.028 | 0.037 | 0.033 |
| Verrucomicrobiae | 0.026 | 0.027 | 0.009 |
| Bacilli | 0.028 | 0.019 | 0.013 |
| Fusobacteriia | 0.008 | 0.011 | 0.015 |
| Coriobacteriia | 0.007 | 0.006 | 0.011 |
| Deltaproteobacteria | 0.002 | 0.003 | 0.008 |
| Mollicutes | 0.001 | 0.004 | 0.001 |
| unidentified_Bacteria | 0.000 | 0.001 | 0.003 |
| unidentified_Oxyphotobacteria | 0.001 | 0.001 | 0.000 |
| Other | 0.001 | 0.001 | 0.000 |
| Order |  |  |  |
| Clostridiales | 0.444 | 0.512 | 0.544 |
| Bacteroidales | 0.141 | 0.120 | 0.171 |
| Selenomonadales | 0.122 | 0.107 | 0.118 |
| Bifidobacteriales | 0.103 | 0.086 | 0.029 |
| Pasteurellales | 0.048 | 0.044 | 0.024 |
| Erysipelotrichales | 0.028 | 0.037 | 0.033 |
| Verrucomicrobiales | 0.026 | 0.027 | 0.009 |
| unidentified_Gammaproteobacteria | 0.035 | 0.015 | 0.009 |
| Lactobacillales | 0.028 | 0.019 | 0.013 |
| Fusobacteriales | 0.008 | 0.011 | 0.015 |
| Coriobacteriales | 0.007 | 0.006 | 0.011 |
| Enterobacteriales | 0.004 | 0.005 | 0.004 |
| Desulfovibrionales | 0.002 | 0.003 | 0.008 |
| Actinomycetales | 0.002 | 0.002 | 0.004 |
| Other | 0.004 | 0.009 | 0.011 |
| Family |  |  |  |
| Lachnospiraceae | 0.209 | 0.275 | 0.285 |
| Ruminococcaceae | 0.193 | 0.211 | 0.233 |
| Bacteroidaceae | 0.117 | 0.095 | 0.139 |
| Bifidobacteriaceae | 0.103 | 0.086 | 0.029 |
| Veillonellaceae | 0.055 | 0.068 | 0.068 |
| Acidaminococcaceae | 0.067 | 0.039 | 0.050 |
| Pasteurellaceae | 0.048 | 0.044 | 0.024 |
| Erysipelotrichaceae | 0.028 | 0.037 | 0.033 |
| Akkermansiaceae | 0.026 | 0.027 | 0.009 |
| Burkholderiaceae | 0.035 | 0.015 | 0.009 |
| Peptostreptococcaceae | 0.029 | 0.016 | 0.015 |
| Streptococcaceae | 0.015 | 0.011 | 0.007 |
| Fusobacteriaceae | 0.008 | 0.011 | 0.015 |
| unidentified_Clostridiales | 0.011 | 0.009 | 0.010 |
| Other | 0.066 | 0.065 | 0.083 |
| Genus |  |  |  |
| Not Assigned | 0.329 | 0.334 | 0.289 |
| Bacteroides | 0.117 | 0.095 | 0.139 |
| Bifidobacterium | 0.103 | 0.086 | 0.029 |
| Acidaminococcus | 0.065 | 0.038 | 0.049 |
| unidentified_Lachnospiraceae | 0.021 | 0.037 | 0.056 |
| Dialister | 0.029 | 0.035 | 0.036 |
| Lachnoclostridium | 0.019 | 0.032 | 0.038 |
| Subdoligranulum | 0.016 | 0.020 | 0.044 |
| Blautia | 0.011 | 0.033 | 0.025 |
| Akkermansia | 0.026 | 0.027 | 0.009 |
| Erysipelatoclostridium | 0.014 | 0.024 | 0.024 |
| Romboutsia | 0.028 | 0.015 | 0.014 |
| Comamonas | 0.034 | 0.011 | 0.005 |
| Dorea | 0.007 | 0.017 | 0.020 |
| Other | 0.187 | 0.213 | 0.243 |

Data were expressed as mean.

**Table S3.** Differential abundant bacterial genus between pre_treatment AS patients and HCs.

|  | AS_M0 vs HC | AS_M6 vs HC | AS_M0 vs AS_M6 |
| --- | --- | --- | --- |
| Subdoligranulum | *** | * | NS |
| Bacteroides | *** | NS | NS |
| Bifidobacterium | ** | NS | NS |
| Streptococcus | *** | *** | NS |
| Megamonas | ** | * | NS |
| unidentified_Erysipelotrichaceae | *** | *** | NS |
| unidentified_Prevotellaceae | *** | NS | NS |
| Romboutsia | *** | * | NS |
| Blautia | *** | *** | NS |
| Lachnoclostridium | *** | NS | * |
| Lachnospira | *** | NS | * |
| Phascolarctobacterium | *** | * | NS |
| Roseburia | *** | NS | NS |
| Alistipes | *** | *** | NS |
| Dorea | *** | *** | NS |
| Holdemanella | *** | NS | NS |
| Tyzzerella | *** | *** | NS |
| Collinsella | *** | NS | NS |
| Parabacteroides | *** | * | NS |
| Erysipelatoclostridium | * | NS | NS |
| Actinomyces | *** | *** | NS |
| Anaerostipes | *** | * | NS |
| Fusobacterium | * | NS | NS |
| unidentified_Clostridiales | * | NS | NS |
| Dialister | *** | NS | NS |
| Parasutterella | ** | NS | NS |
| Fusicatenibacter | *** | *** | NS |
| Megasphaera | ** | NS | NS |
| unidentified_Oxyphotobacteria | ** | NS | NS |
| Lactobacillus | *** | * | NS |
| Sutterella | ** | NS | NS |
| Barnesiella | ** | * | NS |
| Olsenella | * | NS | NS |
| Flavonifractor | ** | * | NS |
| Rothia | *** | *** | * |
| Eggerthella | *** | * | NS |
| Gemella | *** | NS | NS |
| Bilophila | *** | NS | NS |
| Turicibacter | *** | NS | NS |
| Solobacterium | *** | NS | * |
| Desulfovibrio | ** | NS | NS |
| Eubacterium | * | *** | NS |
| Peptoclostridium | * | * | NS |
| Cetobacterium | * | NS | NS |
| Enterococcus | * | NS | NS |
| unidentified_Bacteria | ** | NS | NS |
| Paraprevotella | *** | *** | NS |
| Oscillibacter | *** | * | NS |
| Parvimonas | * | NS | NS |
| Holdemania | * | NS | NS |
| Terrisporobacter | ** | NS | NS |
| Adlercreutzia | *** | * | NS |
| Eisenbergiella | ** | NS | NS |
| Odoribacter | * | * | NS |
| Fournierella | * | NS | NS |
| Weissella | * | NS | NS |
| Atopobium | *** | NS | NS |
| Paraclostridium | *** | NS | NS |
| Lactococcus | ** | * | NS |
| Senegalimassilia | * | * | NS |
| Paeniclostridium | ** | NS | NS |
| Abiotrophia | * | NS | NS |
| Leuconostoc | * | NS | NS |
| Marvinbryantia | ** | NS | NS |
| Oribacterium | ** | NS | NS |
| Oxalobacter | * | * | NS |
| Candidatus_Soleaferrea | ** | NS | NS |
| Anaerotruncus | ** | NS | NS |
| Oscillospira | * | NS | NS |
| Butyricimonas | ** | NS | NS |
| Corynebacterium | ** | NS | NS |
| Thermomonas | ** | NS | NS |
| Coprobacter | ** | NS | NS |
| Angelakisella | ** | NS | NS |

* : *P* <0.05, **: *P* <0.01,***: *P* <0.001, NS: *P* >0.05
